# A Novel Mechanism Underlying the Innate Immune Response Induction upon Viral-Dependent Replication of Host Cell mRNA: A Mistake of +sRNA Viruses' Replicases

**DOI:** 10.3389/fcimb.2017.00005

**Published:** 2017-01-20

**Authors:** Laura R. Delgui, María I. Colombo

**Affiliations:** ^1^Consejo Nacional de Investigaciones Científicas y Tecnológicas, Facultad de Ciencias Médicas, Instituto de Histología y Embriología de Mendoza, Universidad Nacional de CuyoMendoza, Argentina; ^2^Facultad de Ciencias Exactas y Naturales, Universidad Nacional de CuyoMendoza, Argentina

**Keywords:** +RNA viruses, invaginated membranes, double membrane vesicle, TLR3, RIG-I, MDA5, *Semliki Forest Virus*, poliovirus

## Abstract

Viruses are lifeless particles designed for setting virus-host interactome assuring a new generation of virions for dissemination. This interactome generates a pressure on host organisms evolving mechanisms to neutralize viral infection, which places the pressure back onto virus, a process known as virus-host cell co-evolution. Positive-single stranded RNA (+sRNA) viruses are an important group of viral agents illustrating this interesting phenomenon. During replication, their genomic +sRNA is employed as template for translation of viral proteins; among them the RNA-dependent RNA polymerase (RdRp) is responsible of viral genome replication originating double-strand RNA molecules (dsRNA) as intermediates, which accumulate representing a potent threat for cellular dsRNA receptors to initiate an antiviral response. A common feature shared by these viruses is their ability to rearrange cellular membranes to serve as platforms for genome replication and assembly of new virions, supporting replication efficiency increase by concentrating critical factors and protecting the viral genome from host anti-viral systems. This review summarizes current knowledge regarding cellular dsRNA receptors and describes prototype viruses developing replication niches inside rearranged membranes. However, for several viral agents it's been observed both, a complex rearrangement of cellular membranes and a strong innate immune antiviral response induction. So, we have included recent data explaining the mechanism by, even though viruses have evolved elegant hideouts, host cells are still able to develop dsRNA receptors-dependent antiviral response.

## Introduction

Eukaryotic cells are able to detect viruses at multiple steps and benefit from redundant mechanisms with the aim of limiting viral infections. Recognition of viral double-strand RNA (dsRNA) molecules by intracellular Toll-like receptors (TLRs) or retinoic acid inducible gene I-like receptors (RLRs) is a central event which entails the early steps of the immune response elicited during viral infections. A functional anti-viral role of TLR3 has been demonstrated for several animal and human viruses, recently reviewed in Matsumoto et al. (2011). The RLRs family, including the retinoic acid inducible gene I (RIG-I), the melanoma differentiation-associated gene 5 (MDA5) and laboratory of genetics and physiology 2 (LGP2), represent another powerful anti-viral tool parallel to that comprised by TLR3. RIG-I, the leading member of the RLRs, is activated upon dsRNA recognition generating the production of an anti-viral state in the infected-cell and in the surrounding tissue. Its specific activity has been extended reviewed by Kell and Gale (2015). Since we consider TLR3, RIG-I, and MDA-5 as “major sentries” of viral dsRNA molecules, a brief revision of their specific anti-viral action will be presented in this report.

As forced intracellular parasites, viral agents relay on the host cell biosynthetic pathway in order to follow their replication program to generate new viral progeny. Since +sRNA viruses generate dsRNA molecules during their replication process, they have the necessity of hiding their genome from the host cellular dsRNA sentries. So, the induction of specialized membranous niches, often forming organelle-like structures, is a common feature among these viruses. With the aide of pioneering classical electron microscopy (EM) and, during the past few years the most sophisticated electron tomography, several 3-D architecture of viral replication factories have been deciphered (for a technical review on electron tomography, see Frey et al., 2006). Despite several differences among host range, viral structure, genome organization or membrane-donor organelles from the cell, these analyses revealed that +sRNA viruses are able to induce two types of membranous modifications as replicative niches: invaginated vesicles or spherules or a double membrane vesicle type. In this review we will describe, employing a prototype, well-studied, viral agent for each type of membrane alteration, how the virus builds its hideout to shelter from dsRNA receptors.

However, a concern arises when observing that while +sRNA viruses build their replication niches associated to membranes, the host cells are still able to establish an antiviral response, mediated by the cellular receptors that the viruses are intended to hide from. Regarding this important question, a recent proposed mechanism will be included to clarify this intriguing crossroad, a paradigmatic scenario of virus-host cell co-evolution process.

### Toll-like receptor 3: the intravesicular sensor

TLRs are type I transmembrane domain family of proteins with a tripartite structure. They consist of an amino (N)-terminal ectodomain containing leucine rich repeats responsible for ligand recognition, a single transmembrane spanning region and a carboxyl (C)-terminal globular cytoplasmic Toll-like/interleukin-1 (IL-1) receptor (TIR) involved in downstream signaling activation (Gay and Gangloff, 2007). TLRs recognize pathogen-associated molecular patterns (PAMPs) derived from microorganisms and induce an inflammatory response. Due to its ability to recognize dsRNA molecules, the TLR3 is the “cellular major sentinel” against these agents (Thompson et al., 2011). TLR3 recognizes genomic dsRNA or dsRNA replication intermediates present in virus-infected cells independently of the sequence (Alexopoulou et al., 2001). TLR3 is broadly expressed in immune and non-immune cells and has a high level of conservation among vertebrates (Mikami et al., 2012). After synthesis, TLR3 is retained in the endoplasmic reticulum (ER) in unstimulated cells and translocate to the endolysosomal compartment when a dsRNA-stimulation occurs, in a process where the ER membrane protein uncoordinated 93B1 and the leucine-rich repeat containing-protein 59/p34 play an important role (Kim et al., 2008; Tatematsu et al., 2015). Although it has been shown that dsRNA-sensing TLRs are translocated from the ER to lysosomes, upon ligand stimulation, the molecular mechanism of ligand-dependent trafficking of the TLRs is largely unknown. Once within the endolysosomal compartment, TLR3 is able to recognize dsRNA longer than ~40 bp for robust stimulation, largely through the minor groove and the nearby phosphate backbone explaining why recognition is independent of RNA sequence. Genomic nucleic acid material from internalized dsRNA viruses such as reoviruses consisting of long stretches of dsRNA represent molecular structures that are absent in non-infected eukaryotic cells. Endogenous RNAs forming secondary double-stranded structures that are released after necrosis and tissue damage after viral infection represent another source of dsRNA molecules reaching the endosomes, inducing host-derived dsRNA-mediated inflammatory responses through TLR-3 recognition (Kawai and Akira, 2010). However, dsRNA-TLR3 high affinity binding is strikingly dependent on the acidic environment since protonation of histidine on the TLR3 surface is required to allow ionic interaction (Leonard et al., 2008). The dsRNA-TLR3 structure has been recently elucidated revealing that the dsRNA molecules induce the dimerization of TLR3 ectodomain (ECD) inside the vesicle (Liu et al., 2008). Moreover, the proximity of two ECD generates the dimerization of the cytosolic TIR domains (Leonard et al., 2008). Additionally to recognition and dimerization, phosphorylation of the Tyr^759^ and Tyr^858^ residues in the cytoplasmic domain of TLR3 are required for triggering the recruitment of TIR domain–containing adaptor protein interferon-β (TRIF) to the TIR domain of TLR3 (Sarkar et al., 2007). Finally, TRIF recruitment results in stimulation of the transcription factors IRF3 (interferon regulatory transcription factor 3), NF-κβ (nuclear factor-κβ) and AP-1 (activator protein 1) thought two different branches (Alexopoulou et al., 2001; Sato et al., 2003), which finally generates three major antivirals responses: (i) type I interferon production (INF-α/β); (ii) cytopathic effect or infected-cell death; and (iii) generation of pro-inflammatory environment by the activation of NF-κβ and AP-1.

During co-evolution with the cell host, viruses have evolved mechanisms to avoid cell responses against viral infections for their own success. Indeed, hiding dsRNA molecules represents a powerful tool to avoid a harsh cellular war against viruses' replication that initiates after TLR3 recognition and activation.

### RIG-I-like receptors: the cytoplasmic sentries

RIG-I-like receptors (RLRs) mainly include the cytosolic retinoic acid-induced gene I (RIG-I) and the melanoma differentiation-associated gene 5 (MDA5), both sharing the same molecular architecture consisting in a conserved “helicase” core connected to two caspase activation and recruitment domains (CARDs) at the N-terminus, and an RNA binding domain known as C-terminal domains (CTD) (Yoneyama et al., 2005).

In the absence of an RNA trigger, RIG-I is in the cytoplasm in a resting state, in which the CARDs fold back to the C-terminal portion. Upon binding of non-self duplex RNA, RIG-I hydrolyses ATP and undergoes extensive structural rearrangements to reach the fully activated state displaying the N-terminal CARDs and initiating the antiviral signaling cascade (Yoneyama et al., 2004; Fujita et al., 2007; Saito et al., 2007). RNA recognition by RIG-I involves three different RIG-I domains (HEL1, HEL2i, and the CTD) that together clasp the duplex RNA, enwrapping it within a network of interactions that are dominated by polar contacts (Luo et al., 2011). Accurately defining the RIG-I stimulatory RNA structure and sequence remains controversial. However, it seems clear that short (<300 bp) dsRNA panhandle structures are stimulatory if they contain exposed 5′-triphosphate (5′-ppp) and blunted 5′ end (Hornung et al., 2006; Kato et al., 2006; Pichlmair et al., 2006; Marq et al., 2011). An overview of RIG-I interaction with viruses from different genera has been recently reviewed by Kell and Gale (2015).

On the other hand, MDA5 binds to long dsRNA (>1000 bp) with no end specificity (Hornung et al., 2006; Kato et al., 2006; Pichlmair et al., 2006) and by a different mechanism, which leads MDA5 to form a filament along dsRNA, initiated from internal sites in the dsRNA rather than from the ends (Peisley et al., 2011, 2013; Berke and Modis, 2012). Both RLRs form large oligomeric structures around dsRNA molecules that serve as platforms for recruitment and nucleation of mitochondrial antiviral signaling protein (MAVS) (Peisley et al., 2013; Xu et al., 2014). MAVS form a polymeric structure as well, self-propagating drawing soluble monomers from the cytoplasm into the growing polymer (Hou et al., 2011). The polymeric form of MAVS is tethered to the mitochondrial membrane where it triggers the activation of the cytosolic kinases IkB-ε (IKKε) and Tank-binding kinase-I (TBKI), which in turn activate NF-κβ and IRF3, respectively (Fitzgerald et al., 2003; Yoneyama et al., 2004). Activated NF-κβ and IRF3 are translocated into the nucleus where they induce expression of type I interferon and other inflammatory antimicrobial molecules.

Viruses replicate in the host cell to generate new infectious virions. To overcome the innate antiviral response, viral particles include ways to circumvent INF-α/β production achieved by blocking the RLR pathway in its upstream portion to avoid dsRNA-mediated activation of RLRs, further evidencing the potency of such PAMP in triggering a robust innate immune response (Bowie and Unterholzner, 2008). Indeed, hiding dsRNA molecules from RLRs in compartmentalized microenvironments inside the cytoplasm comprise a powerful strategy to tackle RLRs-induced antiviral response.

### *Semiliki Forest Virus*: spherules associated to endolysosomal membranes

Semiliki Forest Virus (SFV) belongs to the *Togaviridae* family, which comprises alphaviruses and the etiologic agent of rubella, the rubella virus. This family is, to date, the sole group of +sRNA viruses that modify endosomal and lysosomal membranes to replicate their genomes (Froshauer et al., 1988; Kujala et al., 1999). Alphaviruses are a genus of viruses generally transmitted by mosquito vectors, which replicate inside the cytoplasm of both, invertebrate and vertebrate cells. They can infect a variety of hosts including small and large mammals, birds, and humans (reviewed by Kuhn, 2013). Among alphaviruses there are several important pathogens affecting human and other animals, including the encephalitogenic alphaviruses that affect horses (e.g., Western, Eastern, and Venezuelan equine encephalitis viruses) and the recently re-emerging chikungunya virus (CHIKV). CHIKV re-emerged in 2004 to cause outbreaks of millions of cases in countries around the Indian Ocean area, in Asia, and recently the Caribbean (http://www.cdc.gov/chikungunya/geo). CHIK causes painful arthritis with symptoms that can persist for years, and can also cause neurological complications and neonatal encephalitis (Schwartz and Albert, 2010). SFV and CHIKV are very similar in terms of molecular and cell biology, e.g., regarding replication and molecular interactions, but are strikingly different regarding pathology: CHIKV is a relevant human pathogen, while SFV is a low-pathogenic model virus, albeit neuropathogenic in mice (Atkins et al., 1999). There are currently no effective vaccines or treatments for human alphavirus infections.

Alphaviruses are small-enveloped particles that enter the cell by clathrin-mediated endocytosis (reviewed by Kielian et al., 2010), followed by fusion of the virus envelope with early endosomal membranes leading to nucleocapsid core delivery into the cytoplasm (Gibbons et al., 2004). The viral nucleocapsid is disassembled with the aide of ribosomes, which have affinity for the capsid protein (Singh and Helenius, 1992). The SFV genome, ~11.5 kb long with a 5′ cap structure and 3′ poly (A) sequence, is translated into a replicase polyprotein, which consists in four non-structural proteins (nsP1–nsP4), involved in viral RNA synthesis, and five structural proteins. The replicase complex [RNA-dependent RNA polymerases (RdRp)] is remodeled by the viral protease nsP2 through sequential cleavages to give rise to the four different units nsP1, nsP2, nsP3, and nsP4 (Vasiljeva et al., 2003). These four units form a macromolecular arrangement responsible of viral genome replication, which also contains RNA originated from newly synthesis (Kujala et al., 2001). However, the RdRp core is formed by nsP4, which harbors a conserved catalytic Gly-Asp-Asp triad (Kamer and Argos, 1984). Together, they give rise to replication complexes (RCs) colocalizing to bulb-shaped membrane invaginations designated spherules (Kujala et al., 2001; Salonen et al., 2003; Spuul et al., 2010). These spherules, thanks to their homogenous size, defined morphology and electron density in infected-cells, were firstly described between late 1960s and early 1970s (Friedman and Berezesky, 1967; Grimley et al., 1968; Friedman et al., 1972). At that time, the spherules were described to have a diameter of ~50 nm and were found located in the membranes of large cytoplasmic compartments, which were termed virus-induced cytopathic vacuole of type *I* (CPV-I) (Grimley et al., 1968). Subsequently, Froshauer et al. demonstrated that the spherules contained endosomal and lysosomal markers and, employing electron microscopy (EM), they observed that the luminal side of the spherule was linked to the cytoplasm by a pore from which electron-dense structures seems to diffuse to the cytoplasm (Froshauer et al., 1988). During the subsequent decades, a great amount of effort has been done to address the biogenesis and dynamics of the CPV-I and, nowadays, a whole picture of the mechanism involved in endosomal and lysosomal membrane modification by SFV has been nicely depicted.

The nsPs are synthesized from the viral positive-sense RNA genome as one polyprotein, which gives rise to four non-structural proteins generated by cleavages catalyzed by nsP2. Of the four nsPs, only nsP1, thanks to an amphipathic helix spotted in the central part of the polypeptide, is the only non-structural protein that interacts with membranes (Peranen et al., 1995; Ahola et al., 1999; Lampio et al., 2000). NsP1 has specific affinity for negatively charged phospholipids explaining its predominant localization to plasma membrane (PM), where those lipids are enriched. The membrane association of nsP1 is mediated through direct interaction of an amphipathic helix with anionic phospholipids and is increased by post-translational palmitoylation of one to three cysteine residues at positions 418–420 (Laakkonen et al., 1996; Ahola et al., 1999). It has recently been demonstrated that nsP1 can only become palmitoylated after associating with membranes via the amphipathic peptide and that this interaction is essential for virus replication (Spuul et al., 2007).

With the aim of following the distribution of SFV RCs Spuul et al. performed double labeling and EM studies in a time course infection (Spuul et al., 2010). The authors discovered that the RCs were predominantly at the PM where numerous typical spherules on the cell surface were observed starting from 1 h post-infection (p.i.), demonstrating that these structures were forming from the PM. From 2 to 4 h p.i., the RCs components were localized to small intracellular vesicles, and then later in the infection, the dsRNA localized to large vacuoles in the perinuclear area, the so called CPV-I. The authors also observed that PM-associated spherules trafficking was strongly dependent upon the activity of class I phosphatidylinositol 3-kinase and a functional actin-myosin network, suggesting that the spherules were an unusual type of endocytic cargo (Spuul et al., 2010). Related to this critical step, these authors, together with others, have previously published, employing EM, the presence of spherules associated to PM-derived vesicles morphologically similar to endocytic vesicles at the stage of internalization (Froshauer et al., 1988; Kujala et al., 2001). Employing a fluorescent recombinant SFV (SFV-ZsG), and LysoTracker stained cells, Spuul et al. observed that the spherules were internalized from the PM in neutral vesicles which underwent several fusion events to be delivered, via a microtubule-based transport, to larger acidic organelles located in the perinuclear area to generate the final stable and static compartment CPV-I, containing hundreds of RCs on their surfaces. The average size of CPV-I reaches 2 μm at 12 h p.i. significantly exceeding the sizes of late endosomes and lysosomes in non-infected cells (Luzio et al., 2007) indicating that alphaviruses have evolved a mechanism to generate and stabilize membranes of the endolysosomal compartment for their replication. A nice graphic schematizing a model for the alphavirus RCs trafficking and biogenesis of CPV-I has been depicted by Spuul et al. (2010).

Members of the *Togaviridae* family, as mentioned above, induce viral replication factories with spherule morphology usurping the endosomal and lysosomal pathway from the cell. Even though no 3-D reconstruction of an alphavirus replication niche has been published to date, spherule structures associated to virus replication has been nicely described for Sindbis Virus (Frolova et al., 2010) and Rubella virus (Fontana et al., 2010), in addition to SFV. The first 3-D reconstruction coming from a +sRNA viral replication niche was published by Kopek et al. (2007). Electron tomography of Flock House Virus (FHV)-infected cells uncovered invaginations or spherules on the external mitochondrial membrane (OMM). Similar to alphaviruses, the spherules detected in FHV-infected cells were about 50 nm in diameter (Miller et al., 2001) and contains a membranous neck with an internal diameter of around 10 nm connecting the spherule lumen with the cytoplasm (Kopek et al., 2007). Contrary to the replication niches of these +sRNA viruses, replication factories of two members of the *Flaviviridae* family, West Nile Virus (WNV) and Dengue Virus (DENV), are derived from the endoplasmic reticulum (ER). Recently, Welsch et al. reported a detailed study deciphering the 3-D architecture of virus-induced membrane rearrangements involved in DENV replication (Welsch et al., 2009). The authors employed several EM techniques including electron tomography (ET) to obtain the 3-D analysis of the virus-induced vesicles revealing that they are invaginations of the ER membrane, connected to the cytosol through a pore that may regulate import of factors required for RNA replication as well as export of newly synthesized genomes to be used for translation or virus assembly. Additionally, and thanks to the powerful ET technique, the authors demonstrated the presence of virus budding sites in close proximity to the pores of replication vesicles, providing for the first time a direct visualization, in 3D, of this process (Welsch et al., 2009). Vesicle formation is probably induced by the non-structural protein 4A (NS4A), which appears to contain a central peripheral membrane domain that intercalates into the luminal leaflet of the ER membrane (Miller et al., 2007).

Regarding plant viruses, even though 3-D structural information on plant +sRNA virus-infected cells is limited, virus-host interactions have been extensively studied for Brome Mosaic Virus (BMV), a member of the *Bromoviridae* family, or the Beet Black Scorch Virus (BBSV), a member of the *Tombusviridae* family, both generating convolution and invagination of the ER membrane and neck-like channels connecting the interiors of spherules to the cytoplasm to replicate inside (Bamunusinghe et al., 2011; Cao et al., 2015).

### Poliovirus: double-membrane, autophagosomal-like vesicles ER-associated

Polioviruses belong to the genus Enterovirus of *Picornaviridae* family. Viruses in this family have nonenveloped particles with a tightly packaged, non-segmented, single-stranded, ssRNA. Among its many members are numerous important human and animal pathogens, such as poliovirus, hepatitis A virus, foot and mouth disease virus (FMDV), enterovirus 71, and rhinovirus (Racaniello, 2013). Poliovirus particles, as other members of the family, consist of an icosahedral protein shell surrounding the naked RNA genome of around 7500 nucleotides. The basic building block of the picornavirus capsid is a protomer, which contains one copy each of four structural proteins: VP1, VP2, VP3, and VP4. The shell is formed by VP1–VP3, and VP4 lies on its inner surface (Fry and Stuart, 2010). The viral ARN encodes a single poliprotein, which is cleaved by virus-encoded proteinases to yield 11–15 final polypeptides. The polyprotein contains three regions: P1, P2, and P3. The P1 region encodes the viral capsid proteins, whereas the P2 and P3 regions encode proteins involved in protein processing and genome replication (Stanway, 1990).

The initial attachment of the virion to the host cell plasma membrane involves the receptor CD155, a type I transmembrane protein member of the immunoglobulin superfamily of proteins (Mendelsohn et al., 1989), causing a conformational change in the capsid which leads to viral internalization via a clathrin-independent endocytic process (Fricks and Hogle, 1990; Tuthill et al., 2006). Upon infection, the virus genome replication occurs in the cytoplasm associated to complex membranous replication factories. The first step in genome replication is copying of the positive stranded RNA to form a negative stranded intermediate; this step is followed by the production of additional positive strands (for a revision see Paul and Wimmer, 2015). It is believed that the dsRNA functions as replicative intermediate during the synthesis of viral RNA. A hallmark of this type of virus is the remarkably rearrangement of cellular membranes into organelle-like replicative factories. Interestingly, it has been determined that newly synthetized membranous structures, but not pre-existing cell membranes, are required for viral replication. Thus, the formation of the complex replication factories requires coupled viral translation, lipid synthesis, new membranes generation and viral RNA synthesis (reviewed by Rossignol et al., 2015). Early in the 70's, based on the incorporation of modified lipids into the membranes of poliovirus replication sites (Mosser et al., 1972) indicated that these structures are different from pre-existing membranous compartments, clearly demonstrating that the virus replication factories are “self-tailored” (Mosser et al., 1972). It was shown that several poliovirus and host proteins are involved in the membrane rearrangements that are essential for virus RNA replication (reviewed by Jackson, 2014). To explore the role of individual viral proteins, cells were transfected or microinjected and visualized by electron microscopy to study the complex cellular changes that take place during viral infection. The viral protein 2BC (a P2 proteolytic precursor of 2B and 2C proteins) is responsible for the generation of 50–350 nm clusters of empty vesicles limited by a single membrane, usually in peripheral regions of the cell containing a high concentration of the 2C epitope (Suhy et al., 2000). In contrast, when 2BC and 3A proteins are expressed, membrane vesicles that displayed double membranes, cytoplasmic luminal contents, and substantial immunolabeling by anti-2C antibody were observed resembled those observed during poliovirus infection and consistent with the idea of an autophagic origin for these membranes (Schlegel et al., 1996; Suhy et al., 2000, please, see below).

Regarding the participation of host proteins it was found that the Golgi-resident small G protein Arf1 (ADP-ribosylation factor), as well as its activator GBF1 [a guanine nucleotide exchange factor (GEF)], were recruited to sites of poliovirus replication (Belov et al., 2005). It is well known that Arf1 is critical for the proper functioning of the secretory pathway, thus, its translocation toward the viral replication complexes may account for the inhibition of protein secretion observed in infected-cells (for a revision see Belov et al., 2007). In addition, it was shown that the release of COPII-coated vesicles that bud from ERES (ER exit sites) increases in poliovirus-infected cells (Trahey et al., 2012), and that expression of a dominant negative mutant of the ER-resident GTPase Sar1confirmed that PV requires functional ER exit sites for normal levels of RNA production and expression (Hsu et al., 2010). Both observations suggest that the virus replication vesicles may be associated or derived from COPII-vesicles. However, more recent studies indicate that classical COPII vesicles do not seem to be the site for RNA replication, supporting again the idea that specialized “self-tailored” membrane vesicles are involved in the poliovirus factories.

The initial ultrastructural studies of Dales et al. in 1965 showed a marked increase in single membrane vesicles at 3 h p.i. and double-membrane structures associated to viral particles at 7 h p.i., suggesting that autophagic vesicles are involved in biogenesis of viral replication factories (Dales et al., 1965). We now know that double-membrane compartments constitute the hallmark vesicles of the constitutive degradative process known as autophagy, or “self-eating” (Schneider and Cuervo, 2014). Autophagy is an essential and constitutive cellular process that regulates turnover of organelles, lipid, and proteins, and plays a role in viral infections (Shi and Luo, 2012). In later studies, Kirkergaard and collaborators were able to identify, using a high pressure freezing and freeze substitution technique, double-membrane structures in infected COS-1 cells at early infection time points (i.e., 4 h p.i.) (Schlegel et al., 1996; Suhy et al., 2000). The virions were in between clustered vesicles and also within double-membrane vesicles labeled with the autophagic protein LC3 where RNA replication was taking place (Belov et al., 2012; Richards et al., 2014). Belov and collaborators performed a three-dimensional analysis showing that indeed the vesicles seemed to be interconnected forming a network of tubular structures (Belov et al., 2012). These recent studies have revealed that the poliovirus factories morphology are indeed complex structures that at early times p.i. consist in clusters of single membrane vesicles, as previously described by Dales et al. (1965) but at later times p.i. most of them are compose by double membrane vesicles and that some of these double membrane structures are not completely closed. These structures may serve to protect double-stranded RNA intermediates during RNA replication (for a comprehensive review see Rossignol et al., 2015).

The Kikergaard's group was the first demonstrating that autophagy benefit poliovirus replication since treatment with autophagy inducers such as rapamycin increased viral particles production (Jackson et al., 2005). In addition, it was also shown that viruses traffic into the mature acidic autophagic vesicles and that maturation of infectious poliovirus particles requires intracellular vesicle acidification (Richards and Jackson, 2012). Cumulative evidence indicate that polioviruses hijack autophagic components to allow their assembly, maturation and exit from the host cell, via a process known as AWOL (Autophagosome mediated exit without cell lysis) (Arita et al., 2012). It has been shown that LC3 silencing with a siRNA leads to a decrease of viral cell-to-cell spread whereas autophagy induction favors this non-lytic release in both cultured cells and mice (Bird et al., 2014). The release of viral particles via a non-lytic process was also previously suggested by a study in the spinal cords of bonnet monkeys (Ponnuraj et al., 1998). In a recent publication it was also shown the release of enwrapped virus via autophagosomal-like vesicles, enriched in phosphatidylserine, which were highly efficient in infection (Chen et al., 2015).

Other +sRNA viruses such as the enterovirus Coxsackievirus (Kemball et al., 2010), Hepatitis C virus (*Flaviviridae* family) (Sir et al., 2012), or Coronavirus such as MVH (Reggiori et al., 2010) also usurp the autophagy pathway and induce remarkably alterations in intracellular membranous components to harbor the sites for viral RNA replication. However, it is important to take into account that significant differences emerge in the mode that different virus hijack cellular components to establish their replication niches (for more comprehensive revisions see Paul and Bartenschlager, 2013; Harak and Lohmann, 2015).

### Mechanism of PRRs-mediated antiviral response associated to +sRNA viruses' replication

**Figure 1:**
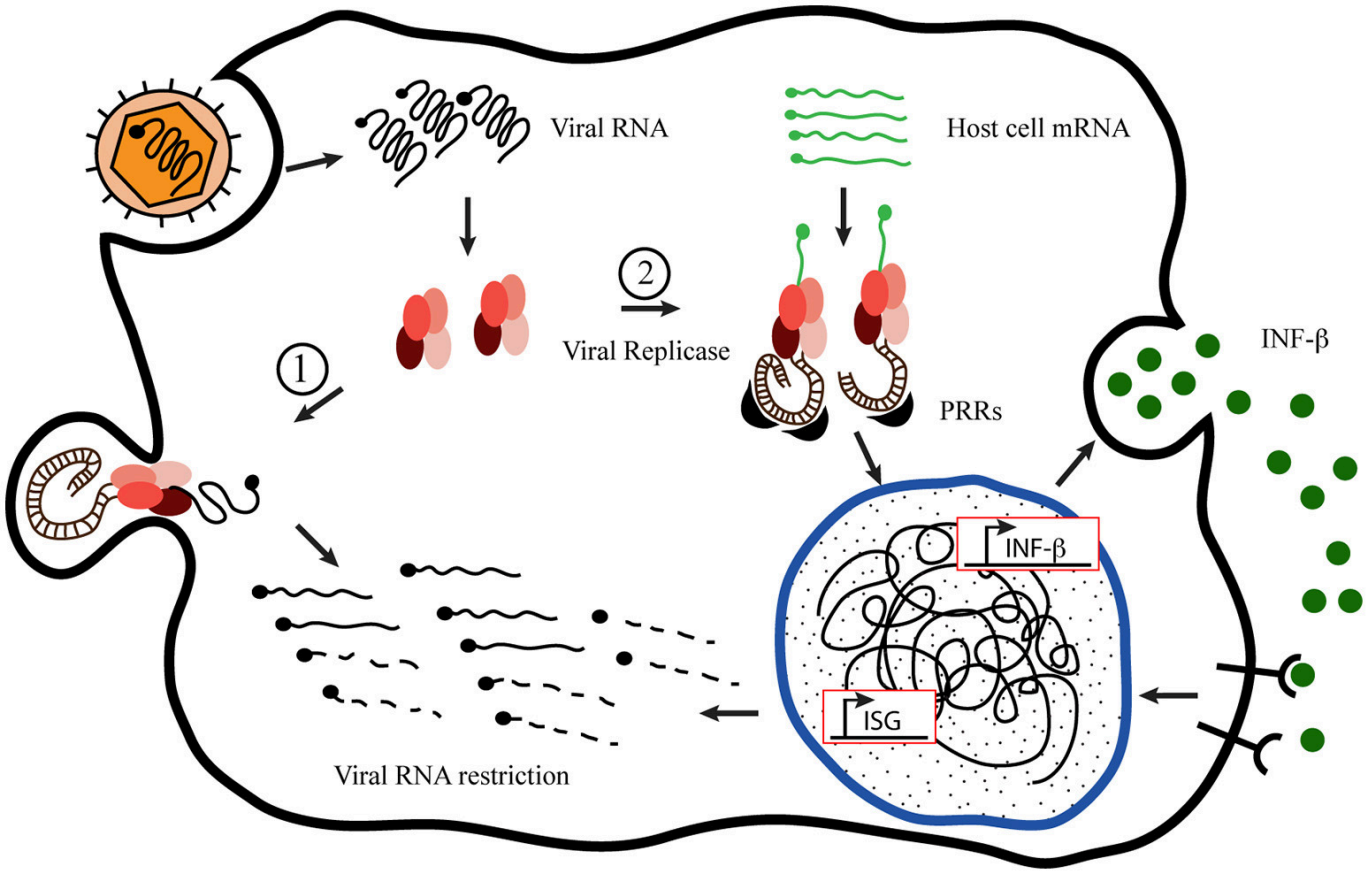
**Proposed model of host-cell mRNA replication by +sRNA viruses' replicases**. Inspired by the “Model of mutant Semiliki Forest Virus replication restriction in fibroblasts,” proposed by Nikonov et al. (2013). After viral internalization and uncoating, the genomic RNA serves as mRNA recognized by the host cell machinery to translate the viral replication complex (RdRp), which binds to the plasma membrane to build up the membranous niche called spherule. Once there, the replicase activity generates new viral genome copies producing double-stranded RNA (dsRNA) molecules as intermediates, which remain hided from the cytoplasmic sentries inside the spherules (1). Nevertheless, in the meantime, the RdRp is able to take cellular mRNA as template originating dsRNA molecules in the cytoplasm, exposed to the Pamp Recognition Receptors [PRRs (MDA-5 and RIG-I)] to initiate the innate immune response, which produces the viral restriction (2).

Using Semliki Forest Virus (SFV) as a prototype to analyze the innate immune response of host-cell to +sRNA viruses, Nikonov et al. (2013) have conspicuously described and proposed a novel mechanism of action for type I interferons induction upon RdPp activity detection inside the cell. Based on previous observations that expression of the viral replicase from others +sRNA viruses, without the replication-competent viral genome, can initiate INF-β promoter; the authors approached their study uncoupling the SFV replicase expression from the viral RNA expression. For achieving this, they generated a plasmid construction bearing the coding sequence of the RdRp from a pathogenic SFV strain, SFV4 pRep. As a control, they generated a plasmid coding an inactive version harboring two changes in the RdRp specific catalytic domain of the nsP4, pRep-GA (Kamer and Argos, 1984). They observed that active SFV replicase is able of inducing INF-β without the replication-competent viral RNA. Employing interfering experiments they demonstrated that RIG-I is the major sensor mediating INF-β induction with MDA-5 acting as an additional sensor, also contributing to INF-β enhancement. Since RIG-I was involved, they suspected that expression of SFV replicase was responsible for generating PAMPs so they characterized these molecules and observed the generation of non-polyadenilated RNA species, larger than 200 nucleotides, containing dsRNA regions and a terminal 5′-phosphate. Moreover, they showed that those dsRNA structures generated by SFV replicase associated to endosomes and lysosomes were the strongest INF-β inducers from those present in the cell, reinforcing the notion that these organelles serve as the sites of SFV replicase docking and viral dsRNA intermediates generation. Based on these and other results obtained through elegant approaches, the authors proposed a novel mechanism of PAMPs generation and INF-β induction by the viral replicase transcription of non-viral host-cell RNA templates. At the same time, the viral replicase molecules would anchor to endosomes membranes to build up membranous spherules where viral dsRNA or 5-ppp RNA intermediates keep inaccessible to host sensors any longer, as suggested by Nikonov and Nikonov et al. (2013), shown in Figure 1.

Accordingly, employing adenoviral vectors as delivery method into murine and human hepatocytes, Yu and colleagues demonstrated that the Hepatitis C Virus (HCV) RdRp NS5B was capable of inducing the innate immune response in the absence of other HCV RNA replication components and/or other non-structural proteins. They further showed that this induction was dependent upon the NS5B enzymatic activity and on the adaptor protein MAVS that functions downstream of RIG-I and MDA5 (Yu et al., 2012).

Supporting this novel mechanism of innate immune antiviral response activation, Painter and colleagues observed that the ectopic expression of RdRp from Theiler's murine encephalomyelitis virus (TMEV, member of the *Picornaviridae* family), in the complete absence of other viral structures, is able to induce ISG activation in an MDA5-dependent fashion. The authors showed the presence of endogenous dsRNA molecules in un-infected tissues of RdRp transgenic mice, which sustained the MDA5-activation. At this point, the authors did not observe a role of RIG-I in this process, but neither rule out this possibility (Painter et al., 2015).

Thus, despite the few +sRNA viruses for which the induction of the innate immune response has been observed to be thanks to RdRp activity, it seems likely that it may be a universal mechanism employed by the host cell to withstand the viral conquest. In this context, since +sRNA viruses conceal the entire replication machinery in membrane-bound cytoplasmic compartments achieved by extensive re-organization of host organelle membranes, it seems to be a matter of time lapsed between virus uncoating with RdRp emergence into the host's cytoplasm and the hideout of the replication machinery.

Finally, and as a consequence, illustrating the virus-host cell co-evolution process once more, virus have evolved with weapons to be protected from innate immune response recognition irrespective of the origin of PAMPs generated during the course of infection.

## Conclusions

For many +sRNA viruses, mentioned throughout this review, RNA replication occurs in association with 50–70 nm diameter membranous vesicles or spherules that form in the lumen of specific cellular organelles, or in double membrane vesicles, reminiscent to that of the autophagic pathway. Although important discoveries on the 3-D architecture of +sRNA virus replication factories have been made, current knowledge is largely descriptive and important information about mechanisms is missing. For instance, the exact topology of RNA replication sites for DMV-type replication factories is yet uncharacterized. Indeed, novel experimental techniques such as metabolic *in situ* labeling of nascent viral RNA and its visualization by employing high resolution and specific microscopy methods will help tackling this important feature of +sRNA viruses replication. Membrane-remodeling events responsible for the biogenesis of replication factories are also mostly unknown. It is likely that a viral protein interplaying with cellular factors might be responsible for that, but precise contributions of individual factors and their temporal and spatial coordination remain to be discovered.

Nevertheless, cellular host is able to develop an innate immune response mediated by PRRs without needing viral replication, which have placed the pressure back on viral agents to evolve specific strategies to counteract its action, while allowing the viral genome to replicate inside their hideouts.

## Author contributions

LD and MC together built up the idea and structure of the review. LD wrote the majority of the text. MC wrote a section of the review and helped performing the revision of the manuscript to its final version.

### Conflict of interest statement

The authors declare that the research was conducted in the absence of any commercial or financial relationships that could be construed as a potential conflict of interest.
